# Telemedical management of severe postpartum hemorrhagic vaginal discharge in a sow – a case report

**DOI:** 10.1186/s40813-026-00501-9

**Published:** 2026-03-23

**Authors:** Alexander Grahofer, Philipp T. Egli

**Affiliations:** 1 Clinic for Swine, Department of Clinical Veterinary Science, Vetsuisse Faculty, University of Bern, Bern, Switzerland; 2 Graduate School for Cellular and Biomedical Sciences, University of Bern, Bern, Switzerland

**Keywords:** Postpartum hemorrhage, Uterine, Farrowing disorders, Puerperal disorders, Oxytocin, Carbetocin, Farrowing pen, Loose housing

## Abstract

**Background:**

Postpartum hemorrhage is a well-recognized and potentially life-threatening complication in both animals and humans. This case report describes a primary postpartum hemorrhage in a second parity sow managed in a free-farrowing system with the support of telemedicine.

**Case presentation:**

A 16 months old Large White sow showed severe hemorrhagic vaginal discharge after the farrowing process. A thorough case history and visual examination of the animal were conducted using telemedical technique prior to treatment. Farrowing began at 14:00 on a Saturday without hormonal induction, and the sow delivered eight live-born piglets by 16:30 without intervention. Overnight, no observations were made. At 04:30 the following morning, the sow was found with nine live-born piglets and one stillborn. During morning feeding, the farmer observed a large amount of blood on the pen walls and severe hemorrhagic vaginal discharge and contacted the veterinarian, initiating a telemedical consultation. Examination revealed a normal appetite, a body temperature of 38.4 °C, firm faeces without visible blood, and no external injuries to the tail, vulva, or vagina. No signs of ongoing labour were observed. Manual palpation of the birth canal was avoided due to the severity of bleeding. Transabdominal ultrasonography revealed no presence of retained piglets. Telemedical evaluation indicated pallor, and the final diagnosis of uterine bleeding was made. Intramuscular administration of 30 IU oxytocin was recommended to stimulate uterine contractions and control the hemorrhage. During the follow up consultation one hour later, the farmer reported that the bloody vaginal discharge stopped within 15 min after oxytocin administration.

**Conclusion:**

This case documents primary postpartum hemorrhage in a free-farrowing sow, a condition not previously reported in the literature. It also illustrates the value of telemedicine in porcine health management, enabling timely assessment and intervention in life-threatening situations.

## Introduction

Postpartum complications in sows pose significant challenges in commercial pig production, with implications for animal welfare, reproductive performance and economic sustainability [1]. Severe haemorrhagic vaginal discharge following farrowing is an infrequent but potentially life-threatening condition that may quickly lead to anaemia, shock, and mortality when treatment is delayed.

Postpartum hemorrhage is a well-recognized and life-threatening complication in other animal species and human obstetrics [2–7]. In human medicine, primary postpartum hemorrhage is defined as a cumulative blood loss of 500–1,000 mL within the first 24 h after delivery and accounts for approximately 96% of all cases of postpartum hemorrhage [2]. In contrast, secondary postpartum hemorrhage occurs after the first 24 h but within 12 weeks postpartum and represents about 6% of cases [2]. Overall, postpartum hemorrhage occurs in approximately 5% of all human births and accounts for about 25% of maternal deaths worldwide following parturition [2, 3]. For animal species, valid data on the incidence of postpartum hemorrhage is lacking. Uterine atony is the most frequent cause, responsible for roughly 70% of cases, followed by obstetrical lacerations (about 20%), retained placental tissue (approximately 10%), and clotting-factor deficiencies (< 1%) in humans [7]. Hence, postpartum uterine contractions are essential for compressing the blood vessels at the placental attachment sites and preventing hemorrhage. In cases of uterine atony, these contractions are weak or absent, leaving the vessels open and predisposing the animal to significant bleeding. Therefore, administration of a hormone that stimulates rhythmic uterine contractions is commonly used to enhance uterine tone and reduce the risk of postpartum hemorrhage [7]. In human and veterinary medicine, treatment typically involves the administration of oxytocin (20–50 IU) or carbetocin (100 µg) to induce sustained uterine contractions [3, 5, 6]. In cases where pharmacological therapy is insufficient, surgical intervention may be necessary to control bleeding in humans [2] as well as in animal species such as bitches, mares, and cows [5, 6, 8]. Conversely, this approach is not used in conventional pig breeding and might only be relevant in miniature and pet pigs.

In potentially life-threatening conditions in swine, prompt recognition and intervention are therefore essential. However, immediate on-farm veterinary presence is not always feasible, particularly in geographically distant herds or when clinical emergencies arise outside routine working hours. In such situations, remote assessment and decision-making become critical for improving animal health and guiding farm personnel through appropriate therapeutic actions. Similar to trends in human healthcare, increasing digitalization is gaining relevance within veterinary medicine [9–12]. In a recent study, pig veterinarians emphasized the potential benefits of digital technologies for health and welfare management [10]. Nonetheless, substantial challenges remain, including consistent and timely data collection, recording, and access, as well as the need for user-friendly, adaptable, and integrated data platforms. Telemedicine, particularly through mobile communication applications, was highlighted by herd veterinarians as a promising tool for remote support [10].

The practical application of telemedicine using video recordings has recently been explored in finishing pigs with outpouching [9]. This study compared video-based clinical assessments with conventional on-farm examinations and demonstrated that agreement between the two methods ranged from poor to almost perfect, depending on the clinical sign assessed and the evaluating veterinarian [9]. Although current legal uncertainties and technological limitations continue to pose barriers to broader implementation [10, 11], the establishment of clear regulatory frameworks and further technological development will likely enable telemedicine to become a valuable component of modern veterinary practice. Therefore, documenting the use of telemedicine in acute, individual clinical cases in porcine health management is essential for advancing digital technologies. Such documentation underscores the value of structured remote clinical support, particularly in situations where timely decision-making may determine survival and influence future reproductive performance.

This case report describes the telemedical management of a multiparous sow experiencing severe postpartum haemorrhagic vaginal discharge, with emphasis on clinical reasoning, communication with farm staff, and outcome evaluation. The case highlights the practical potential of telemedicine as a valuable adjunct to traditional clinical practice in the emergency management of periparturient sow.

## Case presentation

On Sunday morning the Clinic for Swine, Vetsuisse Faculty Bern, received a phone call from a farmer which wanted to have our advice on the following case. A 16-month-old, second-parity Large White sow with an estimated body weight of 230 kg and a body condition score of 3 showed severe hemorrhagic vaginal discharge following the farrowing process. A thorough case history and visual examination of the animal using a video call as telemedical technique was conducted. The breeding farm contains 130 sows and is producing in a one-week batch farrowing system. The farmer mixed their own feed and used a liquid feeding system. Prior to farrowing, the sows were fed a lactation diet supplemented with ground flaxseed twice daily. Five days before farrowing, the sows were moved into free-farrowing pens (dimensions: 2.8 m x 1.8 m) and provided with straw and hay as nesting material.

**Fig. 1:**
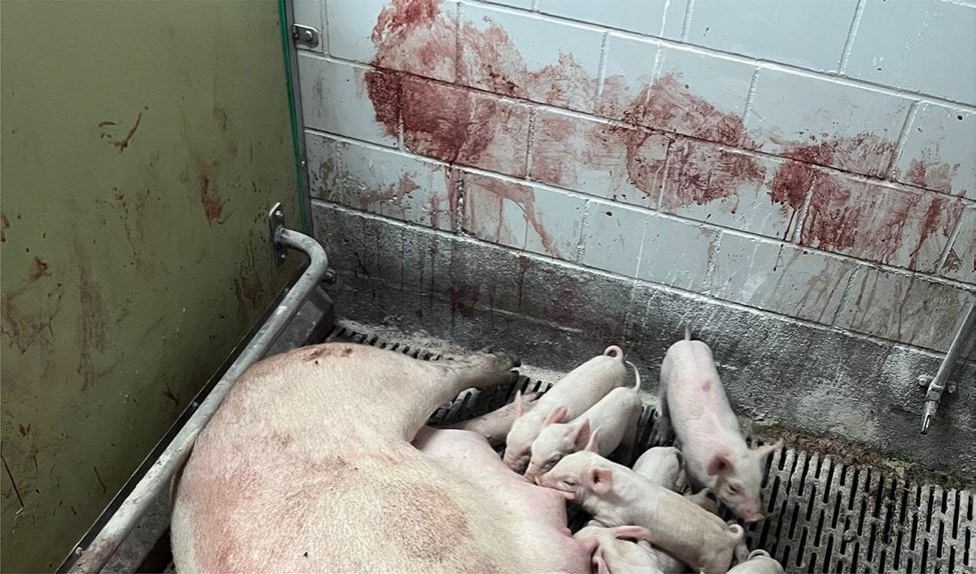
Unusually large amount of blood on the pen walls

**Fig. 2:**
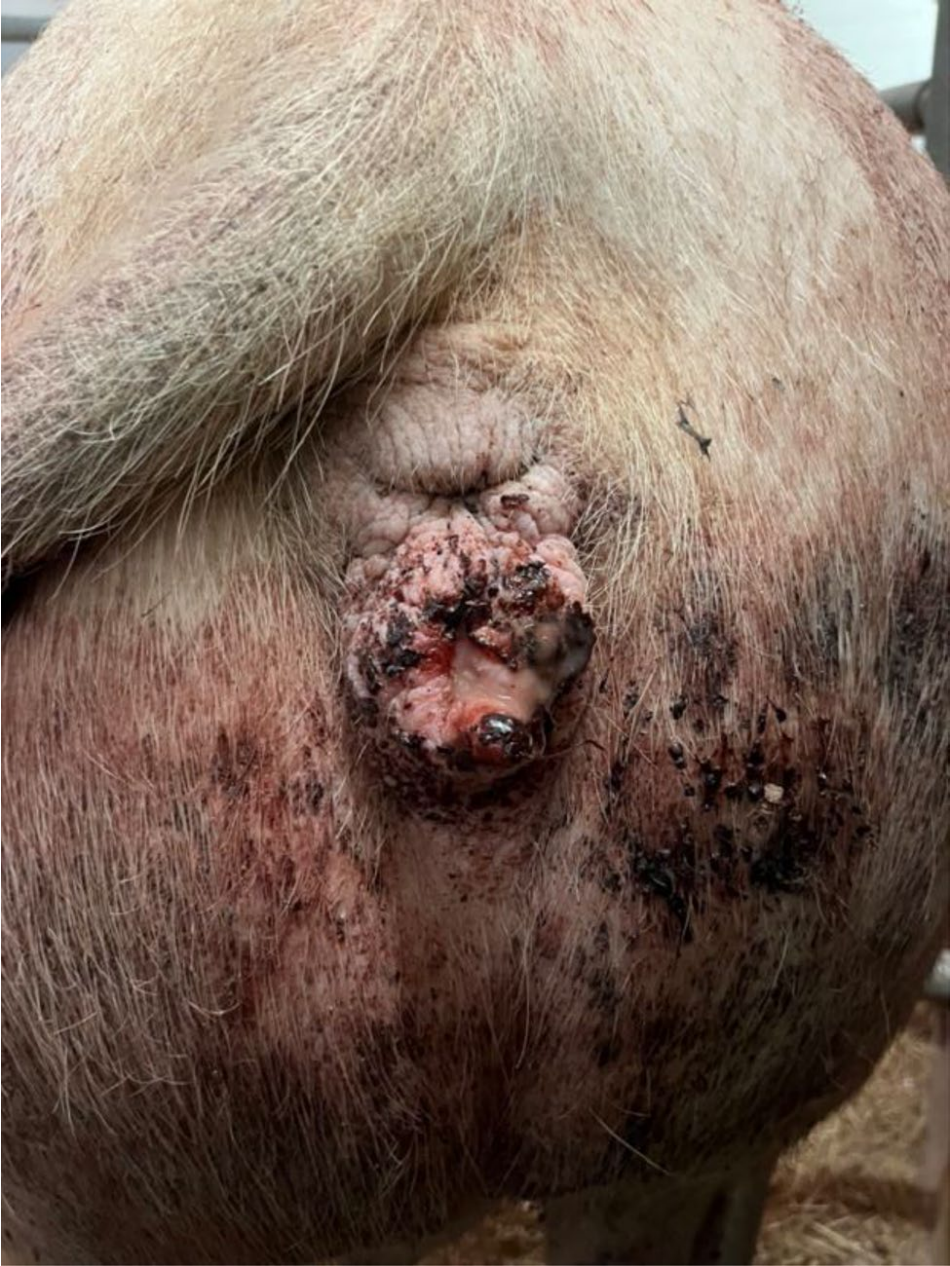
Vulva and vagina revealed no visible injuries

**Fig. 3:**
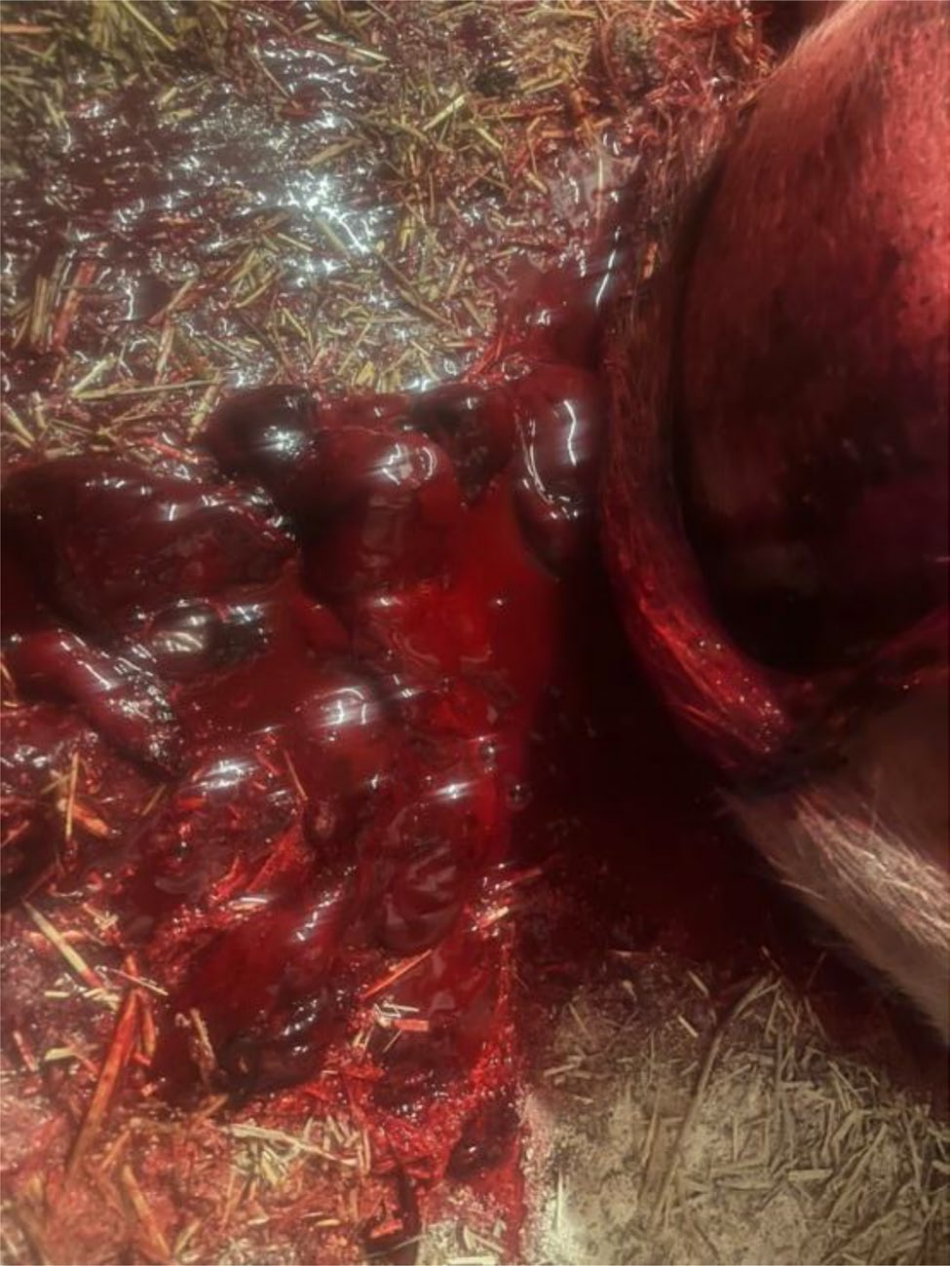
Severe hemorrhagic vaginal discharge

**Fig. 4:**
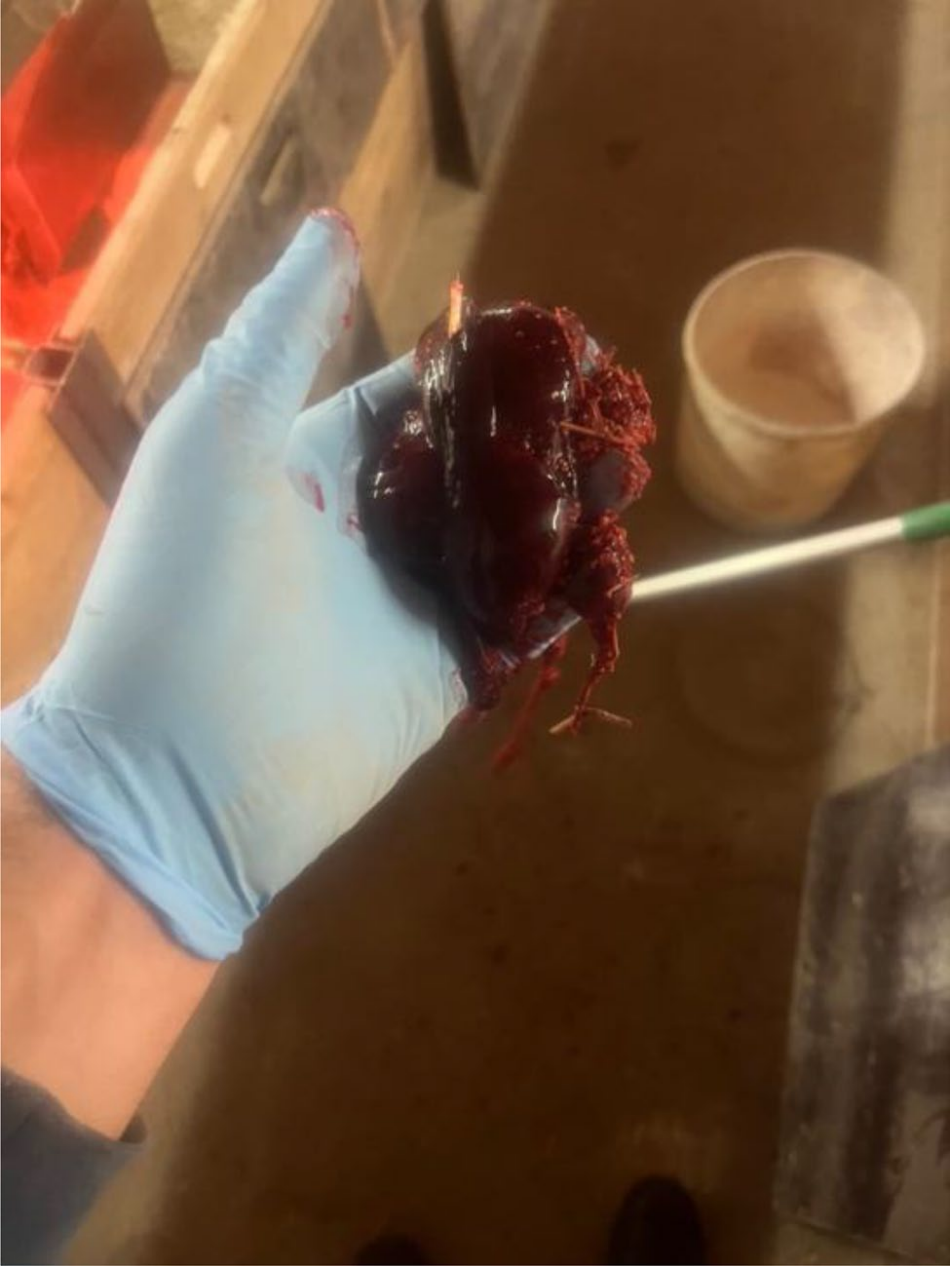
Severe hemorrhagic vaginal discharge

**Fig. 5:**
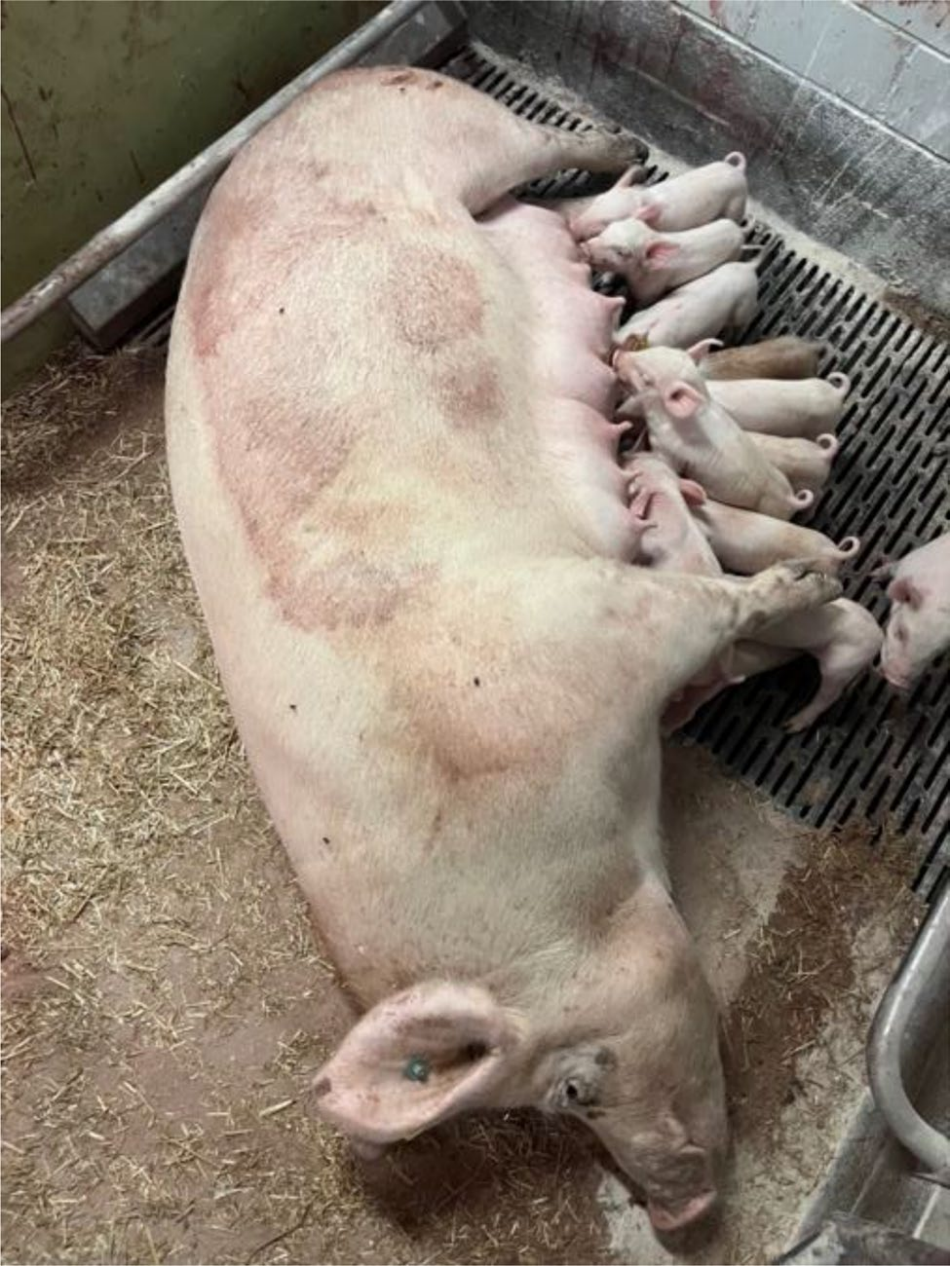
The sow appeared pale

The previous farrowing of the study sow had proceeded on the 118 gestation day without obstetrical assistance and no prolonged farrowing was observed. The sow delivered a total of fifteen piglets, of which fourteen were born alive and one was stillborn. During the puerperal period, the sow developed postpartum dysgalactia syndrome, which was treated with antimicrobial therapy and analgesics. No vaginal discharge was observed at the time of artificial insemination five days after weaning, and no rebreeding was required. The sow remained clinically healthy throughout pregnancy, with no observed disorders. The farrowing process of the sow in question began on Saturday, at 14:00, on the 117. gestation day without birth induction. Continuous monitoring was conducted until 16:30, during which time the sow delivered eight live-born piglets without any obstetrical intervention. No observations were made overnight. On the following morning at 04:30, the sow was found with nine live-born piglets and one stillborn piglet (type II). In addition, parts of the placenta were present in the farrowing pen. In addition, the farmer noticed an unusually large amount of blood on the pen walls (Fig. 1). The sow was carefully examined during morning feeding at 04:30. The sow consumed her entire ration, and had a body temperature of 38.4 °C. The feces were firm, scored as 3 according to the fecal scoring system described by Oliviero [13], and contained no visible blood. Urination was not observed. The farmer cleaned the bloody tail and hind legs to check for possible lesions in this area, but none were detected. Examination of the vulva (Fig. 2) and the caudal part of vagina revealed no visible injuries. While standing, the sow did not exhibit continuous hemorrhagic vaginal discharge. However, after feed intake, she laid down quickly, appeared somnolent, and subsequently showed severe hemorrhagic vaginal discharge (Figs. 3 and 4). The respiratory rate was within the normal range. No signs of ongoing labor (such as abdominal contractions, tail movement, or leg paddling) were observed during the telemedical consultation. The farmer decided not to perform a manual palpation of the birth canal due to the severe bleeding. Instead, the farmer performed a transabdominal ultrasonographic examination with a 3.5MHZ wireless convex ultrasound probe (Nizell Medical GmbH, Lauerz, Switzerland) and a tablet to check for additional piglets in the birth canal. Five ultrasound images and a 30 s video sequence were later reviewed via telemedical consultation. The uterus appeared heterogeneous and was fluid-filled and no additional fetuses were detected within the examined region. Transrectal ultrasonography and rectal palpation were not performed due to the unavailability of the required equipment (linear probe) on the farm and the lack of formal training of the farmer in rectal palpation. The mammary gland was slightly firm, but the piglets were suckling normally. During the telemedical consultation, the farmer was advised to assess the color of the mucous membranes of the eyelids. However, this was not possible, but the telemedical evaluation revealed that the sow body surface appeared pale (Fig. 5). Additionally, the remaining sows in the farrowing batch were systematically evaluated, with no clinical abnormalities.

## Diagnosis

Based on the case history, clinical findings, and evaluation of the environment, a presumptive diagnosis of primary postpartum hemorrhage was established. However, several differential diagnoses must be considered in sows presenting with bloody vaginal discharge after farrowing [14–16]. The first step is to determine the origin of the bleeding, as such discharge may originate from any part of the urogenital tract [14, 15]. Therefore, vulvar or vaginal lesions, as well as farrowing-related complications such as retained piglets or retained placenta, should be excluded. In addition, physiological lochia and bleeding from the umbilical cords of piglets must be considered. Acute cystitis and pyelonephritis may also present similar clinical signs [14, 15]. In some cases, bloody diarrhea can contaminate the vulvar region, giving the false impression of vaginal bleeding. Furthermore, coagulopathy constitutes a differential diagnosis for postpartum hemorrhage in sows.

Hence, several differential diagnoses were considered and subsequently ruled out through telemedical evaluation. Clinical examination revealed no evidence of vulvar or caudal vaginal lesions, thereby excluding these structures as the origin of the hemorrhage. Transabdominal ultrasonography could have identify retained piglets in the uterine horns [17], however, this method was unable to detect piglets lodged in the cervix or birth canal. In this case, direct palpation of the birth canal was avoided to prevent further injury. However, a rectal examination (palpation or ultrasonography) of the cervix would have been required to definitively rule out retained piglets or retained placenta in this case [18–21]. However, due to the lack of appropriate equipment and limitations in on-farm expertise in rectal palpation, this could not be performed. Therefore, this possibility could not be definitively ruled out via telemedical evaluation. Cystitis and pyelonephritis were considered unlikely due to the absence of polyuria and fever. Moreover, pyelonephritis is a condition that typically needs time to develop and would have been expected to manifest with clinical signs before farrowing. The faeces were physiological, thereby ruling out bloody diarrhea as a source of contamination. Finally, coagulopathy was deemed unlikely, as normal clot formation was observed and no other sows were affected. Overall, based on the case history, clinical examination, and additional diagnostic findings, a presumptive diagnosis of primary postpartum hemorrhage was established, although a definitive diagnosis could not be confirmed through the telemedical approach.

## Treatment and outcome

The farmer was informed that telemedical consulting, including visual evaluation of the animal, does not replace an on-farm clinical examination of the animal by a veterinarian on the farm and that primary postpartum hemorrhage was only a presumptive diagnosis, not a definitive one. Since the sow was already exhibiting somnolent behavior, appeared anemic and continued to show severe vaginal bleeding, which is a potentially life-threatening condition for the animal, it was decided in agreement with the farmer to initiate immediate treatment. Since only oxytocin and no carbetocin were available on the farm, in this case the intramuscular administration of 30 IU of oxytocin (Intertocine-S ad us. vet.) was recommended to force strong uterine contraction and therefore stop the bleeding due to contraction of blood vessels. Continuous observation was advised following treatment, with particular attention to the ongoing vaginal bleeding. During the follow up consultation one hour later, the farmer reported that the bloody vaginal discharge stopped within 15 min after oxytocin administration. If this treatment had been unsuccessful, due to an alternative diagnosis, such as a retained piglet, which would have been the only condition altering the treatment decision or preventing cessation of bleeding, euthanasia through the farmer was recommended during the consultation to prevent animal suffering and obtain animal welfare. At the next follow-up on Monday morning, the sow showed a slightly reduced general condition, but demonstrated good feed intake, normal body temperature (38.4 °C) and adequate lactation for her piglets. To estimate the extent of blood loss, hemoglobin concentration was measured on Monday by the farmer using a blood droplet collected from the ear vein and analyzed with a HemoCue Hb 201 device. The affected sow showed a markedly reduced hemoglobin concentration of 42 g/L compared with healthy sows in the same farrowing batch (83, 90, 91, 96, and 110 g/L). These values are also lower than the reported pre-farrowing reference value of 106.2 ± 10.36 g/L from the literature [22], reflecting the physiological blood loss associated with parturition.

## Discussion and conclusion

Postpartum hemorrhage in sows is an uncommon but potentially life-threatening condition that has received limited attention in veterinary literature. The present case highlights both the clinical challenges associated with severe postpartum bleeding in sows and the potential role of telemedicine in acute on-farm decision-making. While postpartum hemorrhage is well-documented in human obstetrics [2, 3], comparable data for swine are scarce, which complicates the establishment of evidence-based treatment protocols.

In this case, the sow presented with severe hemorrhagic vaginal discharge within 24 h of farrowing, consistent with primary postpartum hemorrhage. Differential diagnoses were carefully considered, including retained placenta, uterine atony, vulvar or vaginal trauma, urinary tract pathology, and contamination from fecal blood [2, 3]. Furthermore, it can be speculated that trauma to the birth canal occurred either because of the stillborn piglet, due to an obstructive birth complication, or due to the relatively large body size of the small litter led to postpartum hemorrhage. The absence of visible trauma, retained fetuses, or signs of systemic infection, alongside ultrasonographic evaluation, supported the presumptive diagnosis of secondary uterine atony or trauma to the birth canal leading to hemorrhage. This approach underscores the importance of systematic exclusion of other causes when managing acute postpartum bleeding in sows. Coagulopathy, whether congenital or acquired, represents another vital differential diagnosis for post-partum hemorrhage in sows. Coagulopathy was excluded based on normal blood clotting and the lack of further clinical cases. However, dietary deficiencies, such as inadequate vitamin K, have demonstrated links to coagulopathy, contributing to abnormal bleeding tendencies post-partum [23]. Furthermore, systemic diseases affecting hematological parameters may also provoke an increased bleeding risk, necessitating a comprehensive evaluation of the sow’s health pre- and post-partum.

Immediate intervention is critical in such cases due to the risk of rapid progression to anemia, hypovolemic shock, and mortality. Pharmacological induction of uterine contractions is the mainstay of therapy in humans and other large animal species [3, 5–7]. In the present case, intramuscular administration of oxytocin (30 IU) successfully terminated the hemorrhage within 15 min, demonstrating its efficacy even when applied through telemedical guidance. This aligns with the known uterotonic effect of oxytocin in facilitating uterine contraction and vascular hemostasis. The treatment was conducted with a lower dose of oxytocin than that reported in postpartum cows with hemorrhagic bleeding [5], taking into account that oxytocin levels are higher in free-farrowing sows than in crated sows [19, 23], and to minimize the risk of side effects such as uterine rupture [24]. While carbetocin may offer longer-lasting effects, its unavailability on the farm was the limitation in this case. The hemoglobin measurement obtained the following day confirmed significant blood loss (42 g/L), illustrating the severity of the hemorrhage and highlighting the need for close post-event monitoring [1]. Importantly, if the treatment had not been successful and the animal’s general condition had deteriorated further, euthanasia would have been required in accordance with animal welfare regulations. However, further experience is needed to determine the optimal dosage for cases of uterine postpartum hemorrhage in sows. This case also emphasizes the practical utility of telemedicine in swine health management. Remote assessment enabled rapid visual evaluation, interpretation of ultrasonographic images, and real-time guidance for immediate therapeutic intervention [9–12]. In commercial pig herds, where veterinary presence is not always immediately available, telemedicine may provide a valuable adjunct to ensure timely clinical decisions, potentially improving both animal welfare and reproductive outcomes [9, 10]. Nonetheless, it is important to recognize that telemedical consultation cannot substitute for a thorough on-site veterinary examination in all disease contexts [9]. In this case, the farmer had extensive professional experience in swine production. This background provided a high level of confidence in the farmer’s ability to perform a basic clinical examination and acquire ultrasound images under telemedical guidance. However, it is important to note that such a level of competence cannot be assumed for all farmers, representing a significant limitation of telemedical approaches. Despite the farmer’s experience and training, rectal palpation, which could have provided additional diagnostic information, was not performed due to limited experience with the procedure. Although it can be easily performed using a gloved hand and sufficient lubricant, it should ideally be guided on the farm by a veterinarian during the first attempt [18, 19]. Rectal palpation allows identification of potential causes of birth canal obstruction, such as a hard fecal mass due to constipation, which can then be removed [18, 19]. It also minimizes the risk of vaginal and uterine contamination compared with the vaginal approach and allows assessment of the uterine body and caudal portions of the uterine horns [18, 19]. In addition, rectal palpation could have assessed whether the uterus was capable of contracting in response to mechanical stimulation of the cervix. A further limitation of the telemedical approach was the unavailability of certain equipment on the farm, such as a vaginoscope and a linear probe for rectal ultrasonography. Coagulopathy could not be ruled out without further diagnostic testing, which was also unavailable. Consequently, several differential diagnoses could not be definitively excluded during the telemedical consultation, representing a limitation of this veterinary approach. During an on-farm consultation, rectal palpation of the birth canal and rectal ultrasonography would have been performed by a herd health veterinarian, as it is necessary to confirm a free birth canal prior to oxytocin administration. Furthermore, if piglets were still present in the uterus, uterine contractions induced by a high dose of oxytocin could have disrupted or halted the parturition process, and in severe cases, might have led to uterine rupture. However, the telemedical consultation revealed that immediate intervention was required to control the severe postpartum hemorrhagic vaginal discharge and prevent hypovolemic shock and mortality.

In conclusion, this report illustrates the potential for structured telemedical support in the management of acute, life-threatening postpartum hemorrhage in sows. Early recognition, exclusion of differential diagnoses, and prompt uterotonic therapy were key to the favorable outcome. Future studies are warranted to quantify the incidence of postpartum hemorrhage in sows, evaluate risk factors, and establish standardized treatment guidelines that may integrate telemedicine as an emergency support tool in commercial swine production.

## Data Availability

No datasets were generated or analysed during the current study.
